# *In vitro* studies of *Rickettsia*-host cell interactions: Confocal laser scanning microscopy of *Rickettsia helvetica*-infected eukaryotic cell lines

**DOI:** 10.1371/journal.pntd.0006151

**Published:** 2018-02-12

**Authors:** Stephanie Speck, Tanja Kern, Karin Aistleitner, Meik Dilcher, Gerhard Dobler, Sandra Essbauer

**Affiliations:** 1 Bundeswehr Institute of Microbiology, German Center of Infection Research DZIF Partner, Munich, Bavaria, Germany; 2 University Medical Center Göttingen, Department of Virology, Göttingen, Lower Saxony, Germany; University of Melbourne, AUSTRALIA

## Abstract

*Rickettsia* (*R*.) *helvetica* is the most prevalent rickettsia found in *Ixodes ricinus* ticks in Germany. Several studies reported antibodies against *R*. *helvetica* up to 12.5% in humans investigated, however, fulminant clinical cases are rare indicating a rather low pathogenicity compared to other rickettsiae. We investigated growth characteristics of *R*. *helvetica* isolate AS819 in two different eukaryotic cell lines with focus on ultra-structural changes of host cells during infection determined by confocal laser scanning microscopy. Further investigations included partially sequencing of *rickA*, *sca4* and *sca2* genes, which have been reported to encode proteins involved in cell-to-cell spread and virulence in some rickettsiae. *R*. *helvetica* grew constantly but slowly in both cell lines used. Confocal laser scanning microscopy revealed that the dissemination of *R*. *helvetica* AS819 in both cell lines was rather mediated by cell break-down and bacterial release than cell-to-cell spread. The cytoskeleton of both investigated eukaryotic cell lines was not altered. *R*. *helvetica* possesses *rickA*, but its expression is not sufficient to promote actin-based motility as demonstrated by confocal laser scanning microscopy. Hypothetical Sca2 and Sca4 proteins were deduced from nucleotide gene sequences but the predicted amino acid sequences were disrupted or truncated compared to other rickettsiae most likely resulting in non-functional proteins. Taken together, these results might give a first hint to the underlying causes of the reduced virulence and pathogenicity of *R*. *helvetica*.

## Author summary

The pathogenicity of *Rickettsia helvetica* has not been investigated in depth to date. In humans, seroprevalences up to 12.5% against *R*. *helvetica* have been demonstrated with forest workers being predisposed to infection. However, fulminant clinical cases are rare indicating a rather low pathogenicity compared to other *Rickettsia* species. We therefore investigated growth characteristics of a *R*. *helvetica* tick isolate (AS819) in two different eukaryotic cell lines with focus on ultra-structural changes of host cells during infection as determined by confocal laser scanning microscopy. Further investigations included sequencing of *rickA*, *sca4* and *sca2* genes, which have been reported to encode proteins involved in cell-to-cell spread in some rickettsiae. In contrast to what is known from other rickettsiae, *R*. *helvetica* did not spread directly from cell to cell by actin-based motility presumably due to a deletion in the predicted Sca2 protein. As Sca2 is needed for virulence our results might indicate less virulence and pathogenicity of *R*. *helvetica* isolated from ixodid ticks in Germany.

## Introduction

*Rickettsia* (*R*.) *helvetica* is the most prevalent rickettsia found in *Ixodes* (*I*.) *ricinus* ticks in Germany with varying prevalence up to 17% [1–4]. The organism has mainly been considered non-pathogenic and affected patients usually show a mild disease, manifesting in non-specific fever without erythema (so-called uneruptive fever), headache, and myalgia [5]. Hence, only a few laboratories in Germany focus on *R*. *helvetica* infection as a differential diagnosis and infections might be underdiagnosed due to mild symptoms in most cases. The latter might be the reason that, to the authors’ best knowledge, no clinical case in humans has been reported from Germany so far. However, more severe clinical cases have been demonstrated in Sweden including septicemia [6], myocarditis [7] and meningitis [8]. Only recently, complex phylogenetic studies showed that *R*. *helvetica* phylogenetically was misplaced in the spotted fever group (SFG) [9]. In contrast, *R*. *conorii* subsp. *conorii*, a typical representative of the SFG, causes Mediterranean spotted-fever, a disease that is characterized by fever, an eschar at the site of the tick bite, and a rash spreading to the palms and soles. The disease is endemic in southern Europe but single cases have also been reported from the central and northern European mainland [10].

As many obligate intracellular bacteria, rickettsiae proliferate in the cytoplasm as dispersed, individual bacteria but may also occasionally be found in clusters and in the nucleus [11]. The primary targets for rickettsiae during infection are endothelial cells of the middle and small vessels [12–13]. Disseminated infection of the endothelium and subsequent pathophysiological effects lead to most of the clinical characteristics described for rickettsial diseases [13]. Rapid spread within host tissues is a crucial step in many infectious diseases [14]. For some *Rickettsia* species it has been proven that rickettsiae harness the host cell actin cytoskeleton for intracellular movement and cell-to-cell spread. This phenomenon was first reported by Teysseire et al. [15] and Heinzen et al. [16] who observed associations between *R*. *conorii* and *R*. *rickettsii* and host F-actin. Most of the SFG rickettsiae (SFGR) as well as *R*. *typhi* assemble actin tails and undergo actin-based motility mediating cell-to-cell spread and enhancing virulence [17–18]. Two actin-polymerizing proteins have been identified in SFG rickettsiae: RickA, which activates the actin-related protein-2/3 (Arp2/3) complex of the host [19–20], and surface cell antigen 2 (Sca2) which has been suggested to mimic eukaryotic formin proteins [18,21]. Cardwell and Martinez [22] identified the minimal domain within the Sca2-protein of *R*. *conorii* that is sufficient for stimulating actin polymerization. Most recently, Sca4 was identified as a secreted effector of spread independent from actin-based motility in the SFG *R*. *parkeri* [23].

So far—to the authors’ knowledge—only a single study on growth characteristic of *R*. *helvetica* in eukaryotic cells exists [24] besides the original description of *R*. *helvetica* in 1993 [25]. Based on host cell decomposition and intranuclear growth of *R*. *helvetica*, the study by Elfving et al. [24] underlined the pathogenic ability of *R*. *helvetica*. Here, we report growth characteristics of *R*. *helvetica* isolate AS819 in two different eukaryotic cell lines with focus on ultra-structural changes of host cells during infection as determined by confocal laser scanning microscopy (LSM). Further investigations included sequencing of *rickA*, *sca4* and *sca2* genes, which have been reported to encode proteins involved in cell-to-cell spread in some rickettsiae.

## Materials and methods

### *Rickettsia* isolates and eukaryotic cell lines

Eukaryotic cell lines used in this study were obtained from LGC Standards, Wesel, Germany. L929 cells (murine fibroblasts from connective tissue; up to 25 passages from the original LGC Standards culture) and Vero E6 cells (African green monkey kidney cells; up to 39 passages from the original LGC Standards culture) were grown in Minimum Essential Medium (MEM) supplemented with Gibco GlutaMAX and 1x MEM non-essential amino acids (NEAA) solution (Life Technologies GmbH, Darmstadt, Germany) and 3% fetal calf serum (FCS) at 37°C, 5% CO_2_. The *R*. *helvetica* AS819 isolate (seventh passage from the original culture) used for the growth studies was isolated from *I*. *ricinus*. Species identity was confirmed based on 100% *ompB*-sequence identity (4,848 nt, accession number MF163037) to *R*. *helvetica* type strain C9P9 (accession number AF123725). *R*. *conorii* (Moroccan isolate VR141) and *R*. *honei* (VR1472) were obtained from ATCC, Manassas, USA. All rickettsiae were cultured on Vero E6 monolayers at 32°C in MEM prepared as described above. Flasks were checked daily for detrimental alteration of the monolayer. Rickettsiae were grown until cell layers revealed plaque formation (*R*. *conorii*, *R*. *honei*) or detached from the flasks surface (*R*. *helvetica*). The amount of *R*. *helvetica* in culture supernatants was determined by quantitative *gltA* real-time PCR as described below. A non-infected cell control (MOCK) was carried along with every infection for the evaluation of changes in the cell layers.

### Cultivation and quantification of *Rickettsia helvetica* in two eukaryotic cell lines

L929 and Vero E6 cells (10^5^ cells/well) seeded in 4-well BD Falcon CultureSlides (BD Biosciences, Heidelberg, Germany) were grown to confluent monolayers overnight at 37°C, 5% CO_2_. Prior to infection, monolayers were rinsed with FCS-free medium. Infection was performed with 100 μl of a seed culture containing 5 x 10^5^ *R*. *helvetica* AS819-genome equivalents (corresponding to 5 genome equivalents per cell) determined by quantitative *gltA* real-time PCR as described below. After one hour of incubation at room temperature whilst constantly rocking, wells were filled up with 900 μl MEM supplemented with Gibco GlutaMAX NEAA and 3% FCS. Of every 4-well slide, three wells contained infected cells whilst the remaining served as a non-infected cell control (MOCK). Culture slides were incubated at 32°C, 5% CO_2_. Experiments were performed over a period of 20 days. A slide of each cell line was randomly selected on a daily basis and prepared for the quantification of rickettsia in the cell culture supernatant and the cell layer, respectively. Briefly, cell culture supernatants of each well were harvested individually and stored at -80°C. One ml of cell culture medium per well was added to the remaining cell layers and the slides were frozen at -80°C for one hour. Subsequently, freeze-thawed cells were scraped off, harvested and stored at -80°C until further processing. Nucleic acids were isolated from 200 μl of all samples applying the MagNA Pure LC Total Nucleic Acid Isolation Kit (Roche, Mannheim, Germany) and the MagNA Pure LC 2.0 system (Roche) according to the manufacturer’s instructions. Nucleic acids were eluted in a total volume of 50 μl. Quantification of rickettsiae was carried out by a real-time PCR targeting the single copy citrate synthase gene *gltA* in a Stratagene MX3000P Thermocycler (Agilent Technologies, München, Germany) as described earlier [26–27]. Five μl of nucleic acids were used as a template for the PCR. A commercially available DNA-standard (AmpTech GmbH, Hamburg, Germany) with a defined concentration (2.77 x 10^10^ copies/μl) was used for the quantification of *Rickettsia* genome equivalents. The number of *R*. *helvetica* copies/μl of template was calculated using the Stratagene Software by comparing the samples to a serial dilution of the DNA-standard (2.77 x 10^5^ to 2.77 x 10^1^ copies/μl).

### Preparation of cell lines for confocal laser scanning microscopy (LSM)

Cells were seeded in μ-Slide 8 well ibiTreat chambers (ibidi GmbH, Martinsried, Germany). Briefly, 2.5 x 10^5^ cells/well were grown to near confluence overnight and were infected with 5 genome equivalents per cell as described above. After an initial incubation period of 1 h at room temperature, slides were transferred to 32°C, 5% CO_2_. Fixation and staining procedures were performed at room temperature. At 24 hours-intervals, culture supernatants were discarded and cells were washed using phosphate-buffered saline (PBS, pH 7.2) pre-heated to 37°C. Cells were fixed for 15 min in methanol-free formalin (3.7%). Following a wash with PBS, cells were permeabilized with 0.1% Triton X-100 in PBS for 4 min. To reduce nonspecific binding and background signal, blocking was performed in PBS with 1% bovine serum albumin for 60 min. Thereafter, cells were incubated with an anti-SFG *Rickettsia* antibody (1:2, Fuller Laboratories, Fullerton, USA) for 30 min. After washing with PBS, a mouse monoclonal anti-alpha-tubulin antibody (1:200, Molecular Probes, Life Technologies) was added for 30 min and F-actin was stained with Alexa Fluor 568 Phalloidin (1:100, Molecular Probes, Life Technologies) in parallel. Samples were washed four times and the secondary antibody for *Rickettsia* staining, an Alexa Fluor 647-labeled goat anti-human IgG (1:2000, Molecular Probes, Life Technologies), was added for 30 minutes. In addition, Alexa Fluor 488-labeled goat anti-mouse IgG (1:2000, Molecular Probes, Life Technologies) was added for staining of microtubuli. Washing another four times was followed by nuclei counterstaining with DAPI (4'.6-Diamidino-2-Phenylindole, Dilactate, 1:5000, Molecular Probes, Life Technologies). Confocal images were obtained with a Zeiss LSM 710 using an EC Plan-Neofluar 40x/1.30 Oil DIC M27 objective and using the ZEN software (Zeiss, Jena, Germany). For *R*. *helvetica*-infected cells, staining of slides was performed immediately after fixation each day during the incubation period of 20 d. *R*. *conorii* served as a control but the incubation period was limited to five days after infection due to the rapid cell spread of this rickettsia within cells. Images of L929 or Vero cells infected with *R*. *helvetica* were analyzed using the software Daime [28]. The detection of nuclei and rickettsiae by the software was optimized by comparing manual counts and software-based counts for three pictures each and the following parameters were chosen: Nuclei stained with DAPI were detected using threshold detection for objects larger than 500 pixels. Rickettsiae were detected using edge detection for objects larger than 20 pixels. As it was not possible to visually separate single rickettsiae in highly infected host cells at later time points, we decided to use the signal area instead of the number of objects in order to quantify the infection progress. The signal area corresponding to rickettsiae in at least 450 host cells was analyzed in images from five, ten, fifteen and twenty days *post infectionem* (*p*.*i*.) and the proportion of the area of rickettsiae to the area of cell nuclei was calculated for each time point.

### Plaque assay

Confluent L929 and Vero E6 cells grown in 6-well plates were washed two times with FCS-free Medium. One ml of *R*. *helvetica* culture supernatant (undiluted and serially diluted 10^−1^–10^−4^) was added per well. One well contained an uninfected control. Plates were incubated at room temperature for 1 h while constantly rocking. Double concentrated M199 (Gibco Thermo Fisher Scientific, Waltham, USA) containing 10% FCS was mixed with an equal volume of pre-heated 2% Agarose (Agarose LE, Biozym, Hessisch Oldendorf, Germany) and each well was then filled with 4 ml of agarose overlay. Plates were incubated for 21 d at 32°C, 5% CO_2_. Formalin (3.7%) fixation (room temperature, 1 h) was done on days 7, 14, and 21 *p*.*i*.. After removal of the agarose plugs the cells were stained using crystal violet (1%) in 20% ethanol (30 min, room temperature). After staining, the plates were washed and examined. In parallel the BSL-2 Rickettsia *R*. *honei* was used as a positive (i.e. plaque-forming) control.

### Sequence analyses of *rickA*, *sca4* and *sca2* genes and deduced amino acids

Amplification of the partial *rickA* gene was conducted by PCR using primers described by Balraj et al. [29]. PCR conditions were established using DNA from *R*. *conorii* VR141. Direct sequencing of PCR products was carried out by GATC Biotech AG sequencing service (Konstanz, Germany). Determination of open reading frames and subsequent amino acid (aa) alignments with corresponding sequences retrieved from the GenBank database (http://www.ncbi.nlm.nih.gov/) were performed using the software package BioEdit v. 7.2.5 [30]. The BioEdit Sequence Alignment Editor, Version 7.2.5 and the implemented ClustalW, Version 1.4 [31] were applied for sequence analyses. In addition, sequencing results of *rickA* were complemented by data obtained from whole genome pyrosequencing of *R*. *helvetica* AS819 using the Roche 454 GS-FLX platform (data not yet published). 377,211 shotgun reads (135,929,920 bases) were assembled using GS De Novo Assembler version 2.3, GS Reference Mapper version 2.3, and DNASTAR SeqMan Ngen version 10.1.0. On average, the coverage of the *R*. *helvetica* AS819 plasmid was 146-fold and the coverage of the genome was 34-fold. We predicted CDSs using the RAST prokaryotic genome annotation server (http://rast.nmpdr.org/rast). RNAs were identified using tRNAscan-SE v. 1.23 (tRNA), aragorn v. 1.2.34 (tRNA, tmRNA), and RNammer v.2.1 (rRNA). Database searches were done using BLAST and infernal. Hereby *sca2* gene sequences were obtained.

## Results

### Growth kinetics of *R*. *helvetica* AS819 in eukaryotic cell lines

**Fig 1:**
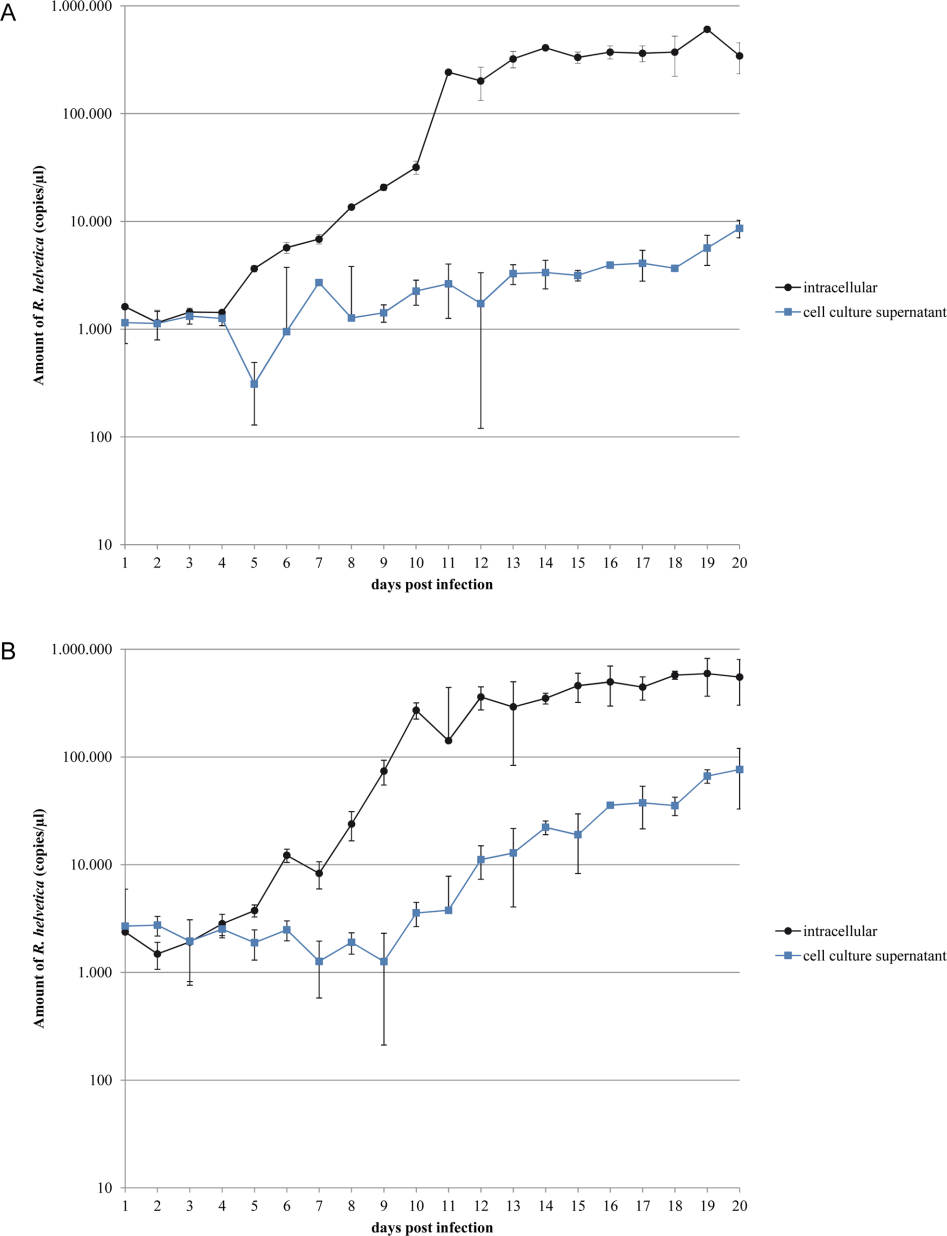
**Growth of *Rickettsia helvetica* AS819 in Vero E6 (A) and L929 (B) cell cultures.** Genome equivalents measured by quantitative *gltA* PCR of infected cell cultures are given at several time points during infection. The values from triplicate infected cultures were averaged; standard deviation error bars are given. Growth studies using Vero E6 (A) and L929 (B) were performed simultaneously. ● indicate *gltA*-copies/μl template-DNA purified from cell layers, and ■ represent *gltA*-copies/μl template-DNA obtained from culture supernatants.

The growth of *R*. *helvetica* AS819 in two eukaryotic cell lines is summarised in Fig 1A and 1B. Results were obtained from triplicates of infected cultures per cell line and time point. After two to four days of lag, a continuous increase of *R*. *helvetica* genome equivalents (given as copies/μl) was seen in both cell lines resulting in a maximum of 6 x 10^5^ copies/μl at day 19. Propagation in Vero E6 cells (Fig 1A) revealed a slight increase of intracellular *R*. *helvetica* DNA copies from day four to eleven after infection. A 100-fold increase was observed from day eleven to day twelve after infection. In L929 cells (Fig 1B), DNA copies of intracellular *R*. *helvetica* tripled from day five to day six and a sharp increase of DNA was seen from day seven to day ten resulting in an approximately 1,000-fold rise. A maximum of 100-fold difference between intracellular and extracellular DNA copies/μl was measured in Vero E6 cells (Fig 1A). In contrast, less than 10-fold difference was found using L929 cell lines (Fig 1B).

### Growth characteristics determined by laser scanning confocal microscopy

**Fig 2:**
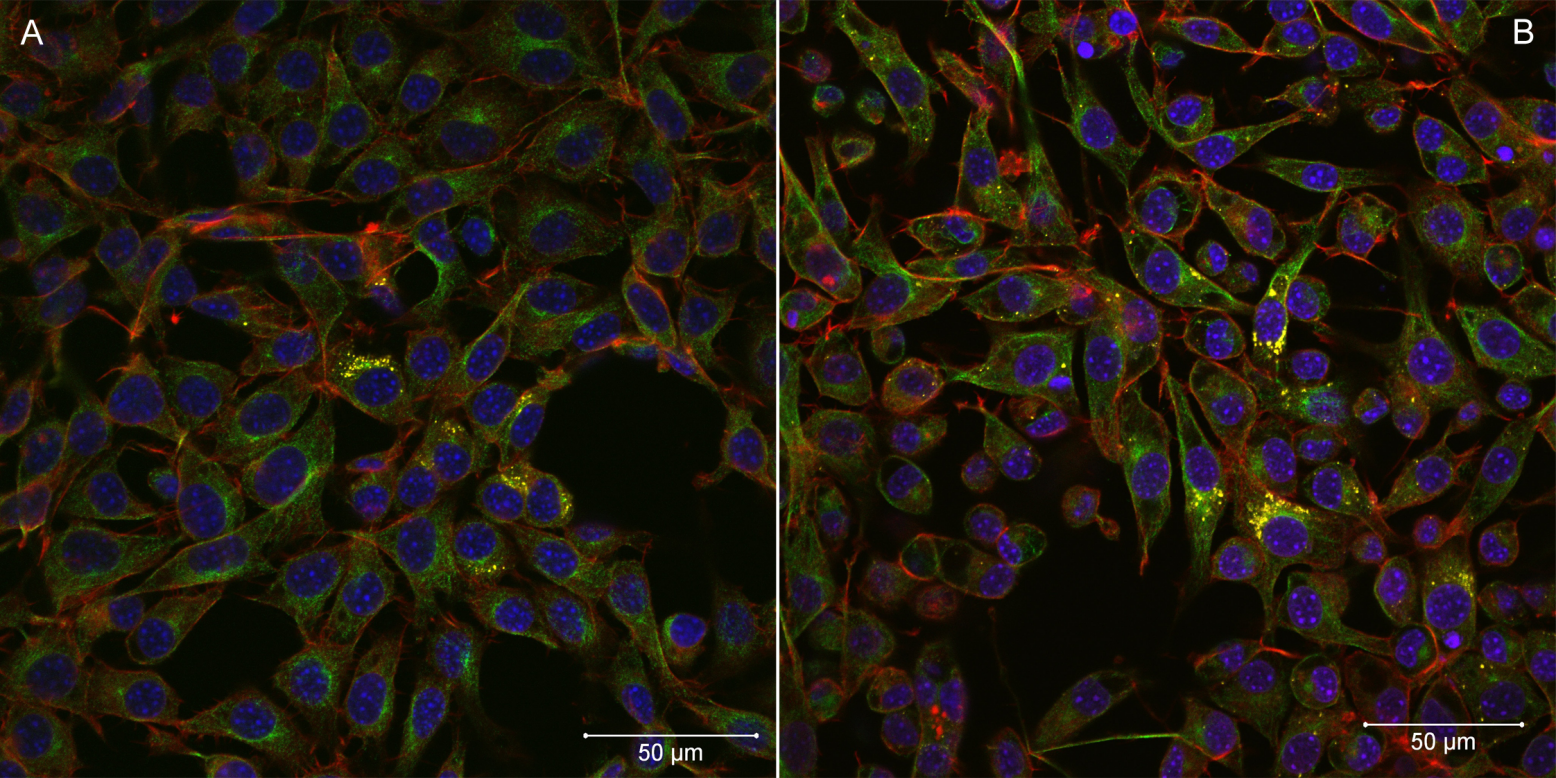
**Laser scanning confocal microscopy of *Rickettsia helvetica* AS819-infected L929 cells five days (A) and ten days (B) after infection.** F-actin was stained with Alexa Fluor 568 Phalloidin (red), α-tubulin appears green (Alexa Fluor 488), nuclei were stained with DAPI (blue), and rickettsiae are coloured yellow (magnification 40x). The bar indicates 50 μm.

**Fig 3:**
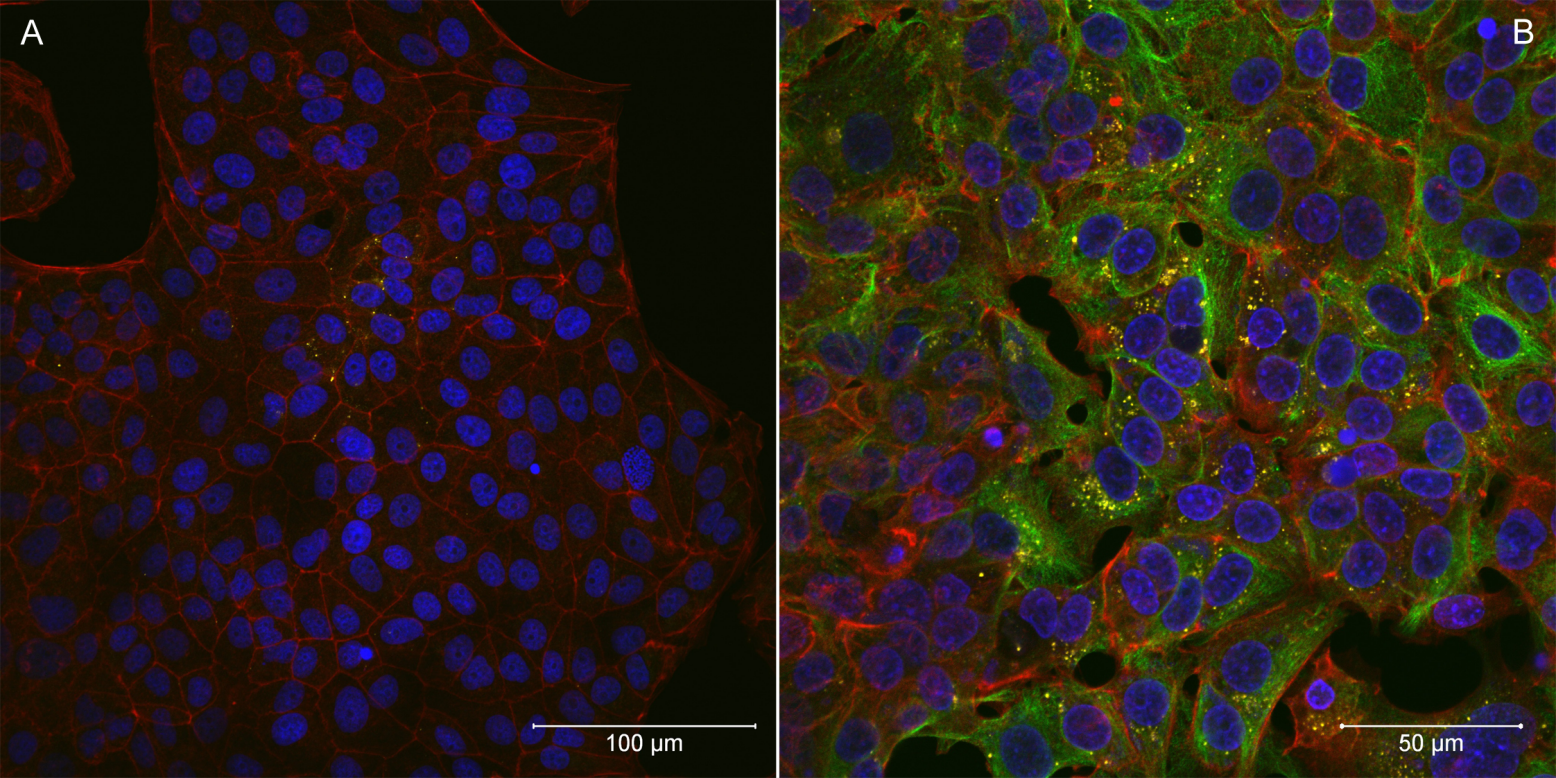
**Laser scanning confocal microscopy of *Rickettsia helvetica* AS819-infected Vero E6 cells five days (A) and 20 days (B) after infection.** F-actin was stained with Alexa Fluor 568 Phalloidin (red), nuclei were stained with DAPI (blue), and rickettsiae are coloured yellow (magnification 40x). The bar indicates 100 μm (a) and 50 μm (b).

Rapid cell-to-cell spread was not seen in *R*. *helvetica* (isolate AS819)-infected cell monolayers. LSM analyses revealed that the percentage of infected cells was rather constant over a period of several days indicating little cell-to-cell spread. Five to ten days *p*.*i*., infected cells were scattered throughout the monolayers (Figs 2 and 3). At that time, up to ten rickettsiae were countable within the cytoplasm of a single cell. From day fifteen until the end of the experiment, the number of infected host cells increased. However, infected cells remained in clusters indicating that *R*. *helvetica* initiated mainly new infection of adjacent cells. *R*. *helvetica* build up in large numbers in the cytoplasm of single cells were visible. No intranuclear bacteria were seen.

**Fig 4:**
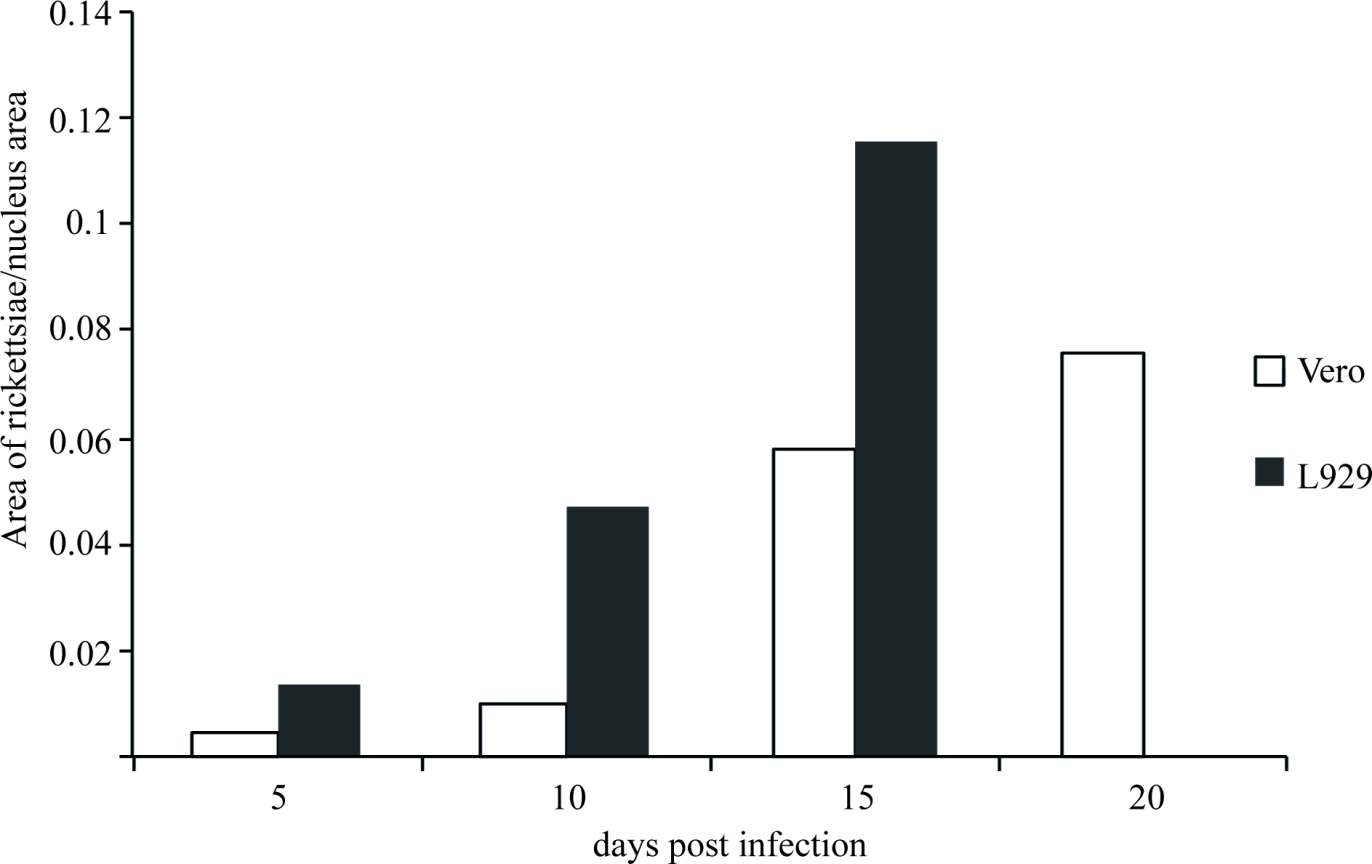
Quantification of rickettsiae per nucleus area over time. The infection progress over time is shown as the increase of rickettsiae per cell nucleus area. For both cell lines a continuous increase of rickettsiae can be observed until day 15 *p*.*i*. After this point in time, L929 cells detached and could not be further investigated while a slight increase was still observable in Vero E6 cells from day 15 to day 20 *p*.*i*. Images with at least 450 host cells were analysed for each time point and cell line.

A quantification of rickettsiae per nucleus area over time revealed higher numbers of *R*. *helvetica* AS819 within L929 cells compared to Vero E6 cells during the whole infection experiment (Fig 4). In both cell lines the number of rickettsiae per cell nucleus doubled from time point to time point until 15 days after infection. The experiment could be pursued until day 20 *p*.*i*. using Vero E6 cells and Fig 4 shows an additional slight increase from day fifteen to day 20 after infection. The detachment of L929 cells increased after day fifteen *p*.*i*., hence, due to the low number of attached cells LSM analyses were impossible after that point in time.

**Fig 5:**
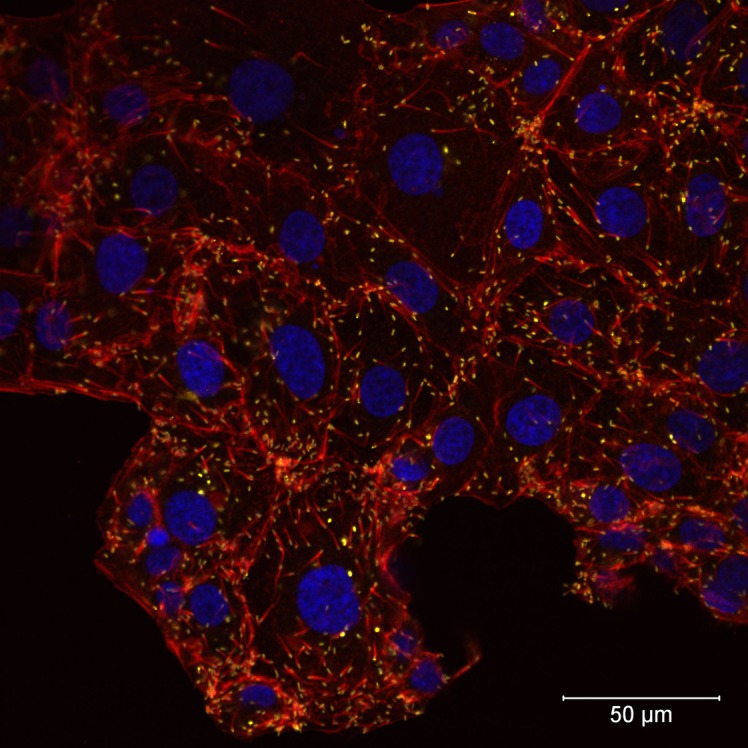
Laser scanning confocal microscopy of *Rickettsia conorii* VR141-infected Vero E6 cells four days after infection. Actin tails at one pole of *R*. *conorii* VR141 can be seen. F-actin was stained with Alexa Fluor 568 Phalloidin (red), nuclei were stained with DAPI (blue), and *R*. *conorii* VR141 is presented in yellow (magnification 40x). The bar indicates 50 μm.

In neither cell line actin polymerization due to *R*. *helvetica* AS819 was detected in contrast to the *R*. *conorii*–infected cell lines (Fig 5). Further, in the latter a prominent plaque formation was visible on day four *p*.*i*. indicating rapid cell-to-cell spread. The analysis of images of the MOCK-infected controls did not yield any signals corresponding to rickettsiae.

### Plaque formation

**Fig 6:**
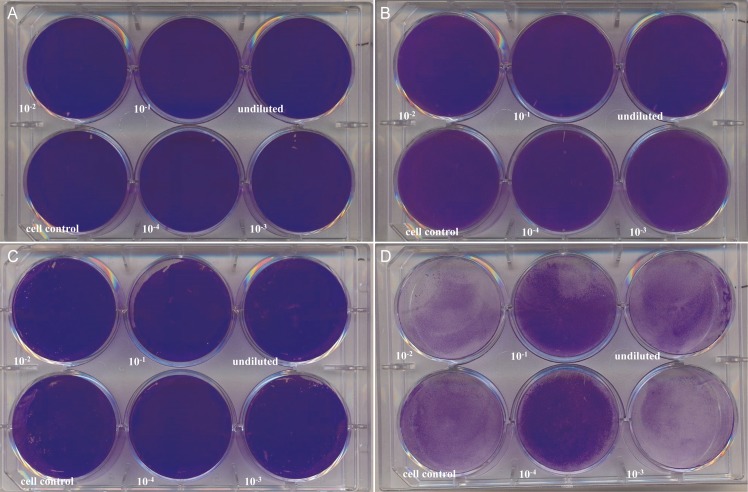
*Rickettsia helvetica* AS819 plaque-forming assay. Vero E6 and L929 monolayers were infected for 21 days with serial dilutions of *R*. *helvetica* AS819. After crystal violet staining, plaques were absent at 7 d *p*.*i*. in Vero E6 (A) and L929 (B) cells as well as after 21 d *p*.*i*. (C, Vero E6; D, L929). Detachment of L929 cells (D) increased with incubation time in agreement with earlier observations.

**Fig 7:**
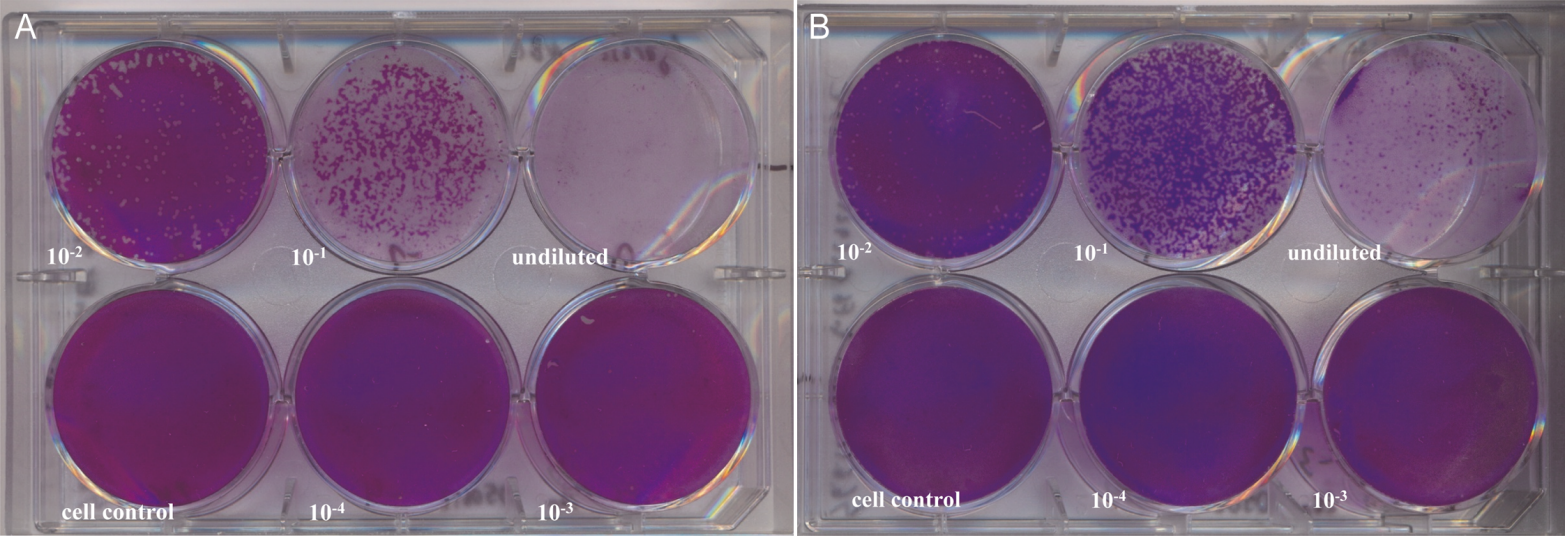
Plaque formation by *Rickettsia honei* in different cell lines. Vero E6 and L929 monolayers were infected for 7 days with serial dilutions of *R*. *honei*. After crystal violet staining plaques were noticed in Vero E6 (A) and L929 (B) cells.

No plaque formation was seen in *R*. *helvetica*-infected cell lines at 7 d *p*.*i*. (Fig 6A and 6B), 14 *p*.*i*. and 21 d *p*.*i*. (Fig 6C and 6D). As noticed before, the detachment of L929 cells increased with prolonged incubation time (Fig 6D). In contrast, plaque formation was readily observed in both cell lines infected with *R*. *honei* at 7 d *p*.*i*. (Fig 7A and 7B).

### Hypothetical RickA, Sca4 and Sca2 in *R*. *helvetica* AS819

The PCR targeting *rickA* and subsequent pyrosequencing resulted in a nucleotide sequence of 1,677 nt (accession number MF163038) with highest similarity (i.e. 90%; 1,528 nt/1,694 nt, 40 gaps) to the complete coding sequence of the *rickA* gene of *R*. *raoultii* strain DnS14 (accession number EU340900). The open reading frame encoded a deduced 559 aa sequence. Using the RAST annotation server, this sequence was identified as hypothetical Wiscott-Aldrich Syndrome Protein (WASP)-like protein.

A protein BLAST search revealed 100% identity (559/559 aa) to a hypothetical protein of the *R*. *helvetica* type strain C9P9 (accession number WP010421970) followed by 83% similarity (465/560 aa) to the Arp2/3 complex-activating protein RickA of *R*. *felis* (accession number WP039594871). Hypothetical RickA in *R*. *helvetica* AS819 revealed an N-terminal domain for binding monomeric actin (G-actin binding site) and several proline (P)-rich repeats were counted within the protein sequence (S1 Fig). In addition, the commonly called WCA region composed of a WASP-homology 2 (WH2) region, a central (C region), and an acidic domain was identified in *R*. *helvetica* AS819. As described for partial RickA of different other *Rickettsia* species, human WASP and N-WASP, a conserved motif (ФXXФXXФXXXRXXФ) was found in the C region (S1 Fig), with Ф representing an aliphatic amino acid, X any residue, and R an arginine [32]. In addition, *R*. *helvetica* AS819 possesses two WH2 regions (S1 Fig) which has also been described for several other rickettsiae.

**Fig 8:**
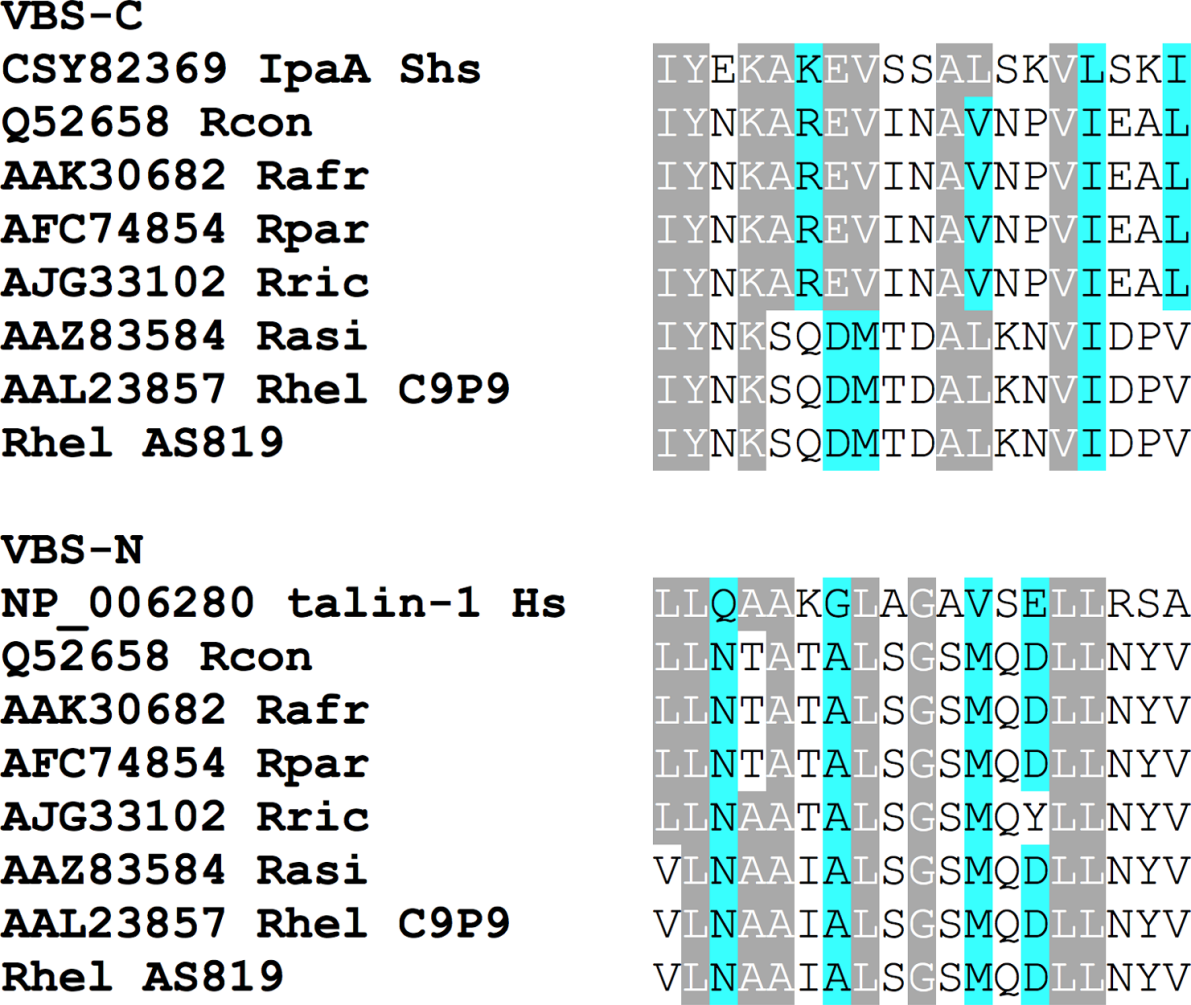
Amino acid sequence alignments of the indicated vinculin-binding-sites. Sca4 of various *Rickettsia* shares significant similarity with vinculin-binding-site (VBS) sequences in mammalian talin-1 or *Shigella* invasion protein IpaA. Gray: aa residues identical to IpaA2 or talin-1; blue: aa residues similar to IpaA2 or talin-1. Abbreviations: Rcon—*R*. *conorii*, Rafr—*R*. *africae*, Rpar—R. *parkeri*, Rric—*R*. *rickettsii*, Rasi—*R*. *asiatica*, Rhel—*R*. *helvetica*, Shs—*Shigella sonnei*, Hs—*Homo sapiens*.

Analyses of nucleotide fragments obtained by pyrosequencing also resulted in 1,917 nt (accession number MF163040) that revealed 97% (1,863 nt/1,918 nt, 13 gaps) similarity to the partial protein PS 120 (D) gene sequence of *R*. *helvetica* type strain C9P9 (accession number AF163009) but 99% (1,901/1,918 nt, 13 gaps) to the partial *sca4* nucleotide sequence of *R*. *asiatica* strain IO-1 (accession number DQ110869). Four partial *R*. *helvetica sca4* gene sequences found in GenBank were identical (767/767 nt, accession number KR150775) or revealed 99% similarity (accession numbers KT825971, KT825970, FJ358501) to the sequence of *R*. *helvetica* AS819. The open reading frame resulted in a 639 aa sequence that shared 95% (599/632 aa, 4 gaps) similarity to the partial protein PS 120 sequence of *R*. *helvetica* type strain C9P9 (accession number AAL23857) and revealed 99% identity (630/637 aa, 4 gaps) to the partial Sca 4 sequence of *R*. *asiatica* strain IO-1 (accession number AAZ83584). Within the aa sequence two stretches were recognized that resemble vinculin-binding sites (Fig 8).

Furthermore, a 4,884-nucleic acid-stretch (accession number MF163039) with highest identity (99%, 4,858/4,888 nt, 7 gaps) to the *R*. *helvetica* C9P9 complete *sca2* gene sequence (accession number AY355375) was obtained. This sequence contains also multiple stop codons and therefore seems to be a pseudogene. Therefore, the predicted aa sequence is disrupted compared to other rickettsiae (S2 Fig) most likely resulting in a non-functional protein.

## Discussion

The pathogenicity of *R*. *helvetica* has not been investigated in depth to date. Its main vector, *I*. *ricinus*, is considered as a generalist with an extraordinarily broad host spectrum including mammals, birds, and reptiles [33]. Hence, potential transmission of *R*. *helvetica* to humans seems likely. In humans, seroprevalences up to 12.5% against *R*. *helvetica* have been demonstrated with forest workers being predisposed to infection [34–37]. However, fulminant clinical cases are rare indicating a rather low pathogenicity compared to other rickettsiae.

There has been only one study describing the life cycle, growth characteristics and host cell response of *R*. *helvetica* in a Vero cell line [24] besides the first description of this species [25]. Concurrent to the results by Elfving et al. [24] we observed a short lag phase of up to four days after infection of the monolayers. However, in contrast to that study [24] *R*. *helvetica* AS819 grew rather slowly. One possible explanation might be that host cell fragments in our seed culture competed with intact host cells for rickettsial attachment thereby decreasing uptake efficiency as has been described for *R*. *prowazeki* seeds [38]. Elfving et al. [24] also reported a lag phase but used suspensions of lysed cells. Moreover, the culture supernatant of our seed culture may have contained an unknown amount of dead or late-growth-phase rickettsia that were no longer in an active growth state [38]. The latter would lead to a lag phase in the intracellular growth [38] as was noticed in our experiments. The percentage of viable *R*. *helvetica* AS819 was not assessed hence, the copy numbers calculated for the seed may have included a large amount of dead organisms. The differences between the Swedish and our study might also be attributed to the different culture techniques used in the respective study: conventional culture in the study at hand versus shell vial centrifugation technique used by the Swedish colleagues [24]. The latter technique has been described to increase infectivity [39] but does not resemble the host-pathogen-interaction during natural infection. No intranuclear *R*. *helvetica* AS819 was detected in the first description [25] and in our experiments, which is in addition in contrast to the study by Elfving et al. [24] and might also result from the centrifugation step used in their study. We showed that *R*. *helvetica* AS819 grew constantly but not rapidly after a phase of adaptation in both tested Vero E6 and L929cell lines, which was confirmed by the LSM investigations. LSM revealed that the dissemination of *R*. *helvetica* AS819 in both cell lines was rather mediated by cell break-down and bacterial release than cell-to-cell spread as has also been described for *R*. *prowazekii* [40]. This was indicated by the irregularly infected cell layers. *R*. *helvetica* AS819 did not produce cytopathogenic effects in Vero E6 and L929 cells which is in agreement with the first description of this species [25]. In addition, actin polymerization due to *R*. *helvetica* AS819 was not detected in both cell lines which has also been described for *R*. *helvetica* elsewhere [25]. This adds to the previous suggestion that *R*. *helvetica* lacks intracellular motility which makes it unlikely to invade nuclei [25,41]. Moreover, plaque formation in Vero E6 and L929 cells was absent after 21 d of infection suggesting that *R*. *helvetica* does not spread from cell to cell. Interestingly, this is in contrast to Rolain et al. [42] who reported formation of small plaques after up to 8 d of incubation using the same cell lines. This difference might be due to strain-specific variation in growth characteristics and virulence as has been described for different strains of *R*. *rickettsii* [43–45]. Moreover, the number of serial passages of cell lines and rickettsia can influence growth characteristics [42–43,46].

RickA-mediated nucleation of actin plays a role in the intracellular spread of some species [16,47]. Although *R*. *helvetica* AS819 possesses a gene encoding for RickA, a bacterial actin nucleator most closely related to WASP/N-WASP-family proteins [19], no host actin polymerization (HAP) was observed. This has also been described for *R*. *raoultii* where *rickA* expression is not sufficient to promote actin-based motility [29]. Furthermore, *R*. *felis* and *R*. *parkeri* possess genes encoding full-length RickA but lack spread by HAP [48]. A disruption of the *rickA* coding sequence as has been shown in *R*. *peacockii* and REIS [49–50] was not confirmed in *R*. *helvetica* AS819 by our sequence analyses. In addition to RickA, Sca2 has been suggested to be necessary for the actin-based motility of rickettsiae [18]. Cardwell and Martinez [22] demonstrated that the first third of the Sca2 passenger domain is highly conserved among SFG rickettsia with the exception of a 39 amino acid deletion. They showed that the deletion of residues 309 to 347 resulted in complete abolishment of actin assembly in *R*. *conorii* [22]. Sca2 aa-sequence analysis from *R*. *helvetica* AS819 revealed a disrupted aa-sequence resulting in a non-functional protein. This is in concordance to the *R*. *helvetica* type strain C9P9. In this strain, *sca2* has been deemed a pseudogene with one or more fragments that don’t span the complete protein [48]. Most likely, this might be responsible for the lack of actin tails observed in *R*. *helvetica* AS819-infected cell lines. Sca2 also seems to play an important role in the initial bacterial-host interaction and Sca2 of *R*. *conorii* mediates both adhesion and invasion of mammalian cells *in vitro* [22]. However, our *R*. *helvetica* isolate AS819 did not exhibit an appreciable defect in adherence or invasion of Vero and L929 cells *in vitro*. This might be attributed to other highly conserved proteins which may compensate for the lack of functional Sca2 [22]. Only recently, Sca4 was identified as another protein from SFG rickettsia that promotes spread [23]. Specifically, Sca4 of *R*. *parkeri* binds to the cell-adhesion protein vinculin and inhibits its activity thereby reducing intercellular tension forces [23,51]. For *R*. *helvetica* isolate AS819 a 639 aa-stretch was identified that revealed 95% similarity to Sca4 of *R*. *helvetica* C9P9. For the latter species Sca4 has been supposed to be a truncated protein [48] which most certainly might also be the case for the strain investigated in this study.

Cell-to-cell spread is one crucial step in the intracellular life cycle of several pathogens including rickettsiae. Actin-based motility contributes to cell-to-cell spread and dissemination within the host. In contrast to other rickettsiae, *R*. *helvetica* isolate AS819 did not spread directly from cell to cell by actin-based motility presumably due to a deletion in the predicted Sca2 protein. As Sca2 is needed for virulence [14] our results suggest less virulence and pathogenicity of *R*. *helvetica* isolated from ixodid ticks in Germany.

## Supporting information

S1 Fig Amino acid sequence alignments of RickA from different rickettsiae and *R. helvetica* AS819. The sequences are trimmed at the end. Red: G-actin binding site; green: WH2 regions; blue: central region; orange: acidic domain. The proline-rich regions are highlighted in grey. A conserved motif can be seen at the end of the sequence, where Ф represents an aliphatic amino acid, X represents any residue, and R is a conserved arginine. Abbreviations: Rhel—*R*. *helvetica*, Rak—*R*. *akari*, Rmas—*R*. *massiliae*, Rrao—*R*. *raoultii*, Rfel—*R*. *felis*, Rcan—*R*. *canadensis*, Rsib—*R*. *sibirica*, Rafr—*R*. *africae*, Rcon—*R*. *conorii*, Rric—*R*. *rickettsii*, Rslo—*R*. *slovaca*, Rmon—*R*. *monacensis*.
(PDF)

S2 Fig Amino acid sequence alignment of the N-terminal third of the passenger domain of Sca2 from different rickettsiae and *R. helvetica* AS819. Numbering above the alignment indicates amino acid numbering according to *R*. *conorii* Sca2 (accession number NP 359747). Dashes within the alignment indicate deletions. * indicate stop-codons. Amino acids 309 to 347, which have been described necessary for the stimulation of actin assembly by Sca2, are shaded in grey; WH2 domains are shaded in blue, and proline-rich domains are boxed in black. Abbreviations: Rcon—*R*. *conorii*, Rric—*R*. *rickettsii*, Rpar—R. *parkeri*, Rpea*–R*. *peacockii*, Rhel—*R*. *helvetica*.
(PDF)
