# Supplementary material for: In vitro studies of Rickettsia-host cell interactions: Confocal laser scanning microscopy of Rickettsia helvetica-infected eukaryotic cell lines
Source: PLoS Negl Trop Dis. 2018 Feb 12;12(2):e0006151. doi: 10.1371/journal.pntd.0006151 (PMC5825168; doi:10.1371/journal.pntd.0006151)
Supplement: S1 Fig — The sequences are trimmed at the end. Red: G-actin binding site; green: WH2 regions; blue: central region; orange: acidic domain. The proline-rich regions are highlighted in grey. A conserved motif can be seen at the end of the sequence, where Ф represents an aliphatic amino acid, X represents any residue, and R is a conserved arginine. Abbreviations: Rhel—R. helvetica, Rak—R. akari, Rmas—R. massiliae, Rrao—R. raoultii, Rfel—R. felis, Rcan—R. canadensis, Rsib—R. sibirica, Rafr—R. africae, Rcon—R. conorii, Rric—R. rickettsii, Rslo—R. slovaca, Rmon—R. monacensis. (PDF) [file pntd.0006151.s001.pdf]

```
                              10        20        30        40        50        60        70        80        90       100
                     ....|....|....|....|....|....|....|....|....|....|....|....|....|....|....|....|....|....|....|....|
Rhel AS819           ------------MAKEIDINKLLAQENNALNTILGQVNKLCEQNKKLQGLIEIQNETKELEKEHNRSLHWFKRFVKTVSNVKYIFIKSEEQLTNEAIKYN
WP_010421970 Rhel C9P9 ------------MAKEIDINKLLAQENNALNTILGQVNKLCEQNKKLQGLIEIQNETKELEKEHNRSLHWFKRFVKTVSNVKYIFIKSEEQLTNEAIKYN
YP_001493700 Rak      ------------MAQEIDINKLLAQENNALNAILSQVNELCTQNKKLQGLIEIQNKTKELAKEHNRSLPWFKRLVKTVSNVKYIFVKSEEQLTNEAIKYN
YP_001499560 Rmas     ---------MYGVAKEIDINKLLAQENNALNTILSQVNELCEQNKQLQGLIEIQNETKELEKEHNRSLPWFKRLVKTVSNVKYIFIKSEEQLTNEAIKYN
ACA30404 Rrao         ---------MYGVAKEIDINKLLAQENNALNTILSQVNELCEQNKQLQGLIEIQNETKELEKEHNRSLPWFKRFVKTVSNVKYIFIKSEEQLTNEAIKYN
YP_246387 Rfel        ---------MYGVAKEIDINKLLAQENNALNTILSQVNELCEQNKKLQGLIEIQNETKELEKEHNRSLPWFKRLVKTVSNVKYIFVKSEEQLTNEAIKYN
YP_001492102 Rcan     MAKETKETKETKETKETDINKLLTQENNALNTILRQVNELCEQNQKLQGVLEIQNEAKELEKEHNRSLPWFKKFVKTVSNVKYIFIKSEEQLTHEAIKHN
ZP_00142939 Rsib      ------------MVKEIDINKLLAQENNALNTILSQVNELCKQNKQLQGLIEIQNETKELEKEHNRSLPWFKRFVKTVSNVKYILIKSEEQLTNEAIKYN
YP_002845437 Rafr     ---------MYGMVKEIDINKLLAQENNALNTILSQVNELCKQNKQLQGLIEIQNETKELEKEHNRSLPWFKRFVKTVSNVKYILIKSEEQLTNEAIKYN
NP_360546 Rcon        ---------MYGMVKEIDINKLLAQENNALNTILSQVNELCKQNKQLQGLIEIQNETKELEKEHNRSLPWFKRFVKTVSNVKYILIKSEEQLTNEAIKYN
YP_001650281 Rric     ------------MVKEIDINKLLAQENNALNAILSHVNELCKQNKQLQGLIEIQNETKELEKEHNRSLPWFKRFVKTVSNVKYILIKSEEQLTNEAIKYN
AEV92458 Rslo         ------------MVKEIDINKLLAQENNALNTILSQVNELCKQNKQLQGLIEIQNETKELEKEHNRSLPWFKRFVKTVSNVKYILIKSEEQLTNEAIKYN
Q9AKP3 Rmon          ------------MTKEIDINKLLAQENNALNTILSQVNELCEQNKQLQGLIEIQNETKALEKEYNRSLPWFKRFVNTVSNVKYIFIKSEEQLTNEAIKYN
                             110       120       130       140       150       160       170       180       190       200
                     ....|....|....|....|....|....|....|....|....|....|....|....|....|....|....|....|....|....|....|
                              G-actin binding site
Rhel AS819           NKVLKDIDNKIYNIAEKSAPLKQELQEEIKKNFEDLTKKNLSKDQRERLSEVFFSYKSKPERFSALHMTNPLQFINAEELEKQYNSLNATKQNIQNLISE
WP_010421970 Rhel C9P9 NKVLKDIDNKIYNIAEKSAPLKQELQEEIKKNFEDLTKKNLSKDQRERLSEVFFSYKSKPERFSALHMTNPLQFINAEELEKQYNSLNATKQNIQNLISE
YP_001493700 Rak      NKVLKSIDNKIYNIAAKSAPLKQELQEEIAKNFKDLTKKDLSKEQRERLSEVYFSYKSKPERFSALHITNPLQFINAEELEKQYNSLNATRQNIQNLISE
YP_001499560 Rmas     NKILKDIDNKIYSIAEKSAPLKQALQAEIEKIFKDLTKKDLSKDQRARVSEVFFSYKSKSERFSALHMTNPLQFINAEELEKQYNSLNATKQNIQNLISE
ACA30404 Rrao         NKILKDIDNKIYNIAEKSAPLKQELQEEIEKNFKDLTKKDLSKDQRERLSEVFFSYKSKPERFSALHMTDPLQFINAEELEKQYNSLNATKQNIQNLISE
YP_246387 Rfel        NKILKDIDNKIYNIAEKSAPLKQELQEEIEKNFKDLTKKDLSKEQRERLSEVYFSYKSKPERFSALNMTNPLQFIKAEELEKQYNSLNATKQNIQNLISE
YP_001492102 Rcan     TKILKDIDNKIYNIVAKSAPLKQALQEEIKKNFEDLTKKDLSKDQRERLAELFFSYKDKPERFSALRMTHPLQFTNAAVLENQYNALNTTKQNIRNLISE
ZP_00142939 Rsib      NKILKDIDNKIYNIAEKSAPLKQALQEEIEKNFKDLTKKDLSKDQRARLSEVFFSYKSKPERFSALHMTNPLQFINAEALEKQYNSLNATKQNIQNLISE
YP_002845437 Rafr     NRILKDIDNKIYNIAEKFAPLKQALQEEIEKNFKDLTKKDLSKDQRARLSEVFFSYKSKPERFSALHMTNPLQFINAEALEKQYNALNATKQNIQNLISE
NP_360546 Rcon        NKILKDIDNKIYNIAEKSAPLKQALQEEIEKNFKDLTKKDLSKDQRARLSEVFFSYKSKPERFSALHMTNPLQFINAEALEKQYNSLNATKQNIQNLISA
YP_001650281 Rric     NKILKDIDNKIYNIAEKSAPLKQALQEEIEKSFKDLTKKDLSKDQRARLSEVFFSYKSKPERFSALHMTNPLQFINAEALEKQFNSLNATKQNIQNLISE
AEV92458 Rslo         NKILKDIDNKIYNIAEKSAPLKQALQEEIEKNFKDLTKKDLSKDQRARLSEVFFSYKSKPERFSALHMTNPLQFINAEALEKQYNALNATKQNIQNLISE
Q9AKP3 Rmon          NKILKDIDNKIYNIAEKSVSLKQELQEEIEKNFKDLTKKDLSKDQRERLSEVFFSYKSKPERFSALHMTNPLQFINAEELEKQYNSLNATKQNIQNLISE
                             210       220       230       240       250       260       270       280       290       300
                     ....|....|....|....|....|....|....|....|....|....|....|....|....|....|....|....|....|....|....|
Rhel AS819           NSNIKELKKIQKQVAEIREEIPYTFFEKLNNIWQNVKNVFVNNSEQVLAKNKESNTRTIRKIDKQLYETKHKFEELIENKERNINDIIAKLPDNEELQKI
WP_010421970 Rhel C9P9 NSNIKELKKIQKQVAEIREEIPYTFFEKLNNIWQNVKNVFVNNSEQVLAKNKESNTRTIRKIDKQLYETKHKFEELIENKERNINDIIAKLPDNEELQKI
YP_001493700 Rak      NSNIKELKEIQKQVAEIREEIPYTFFEKLNNIWQNVKNVFVNNKEKILAKNKESNTRTIRKIDEQLYKTKHKFEELIENKERNINDIISKLPDNEELKNI
YP_001499560 Rmas     NSNIKELKEIQKQVAEIREEVPYTFFEKLNNIWQNVKNVFVNNSEQVLAKNKESNTRTIRKIDEQLYKTKHKFEELIENKERNINDIIAKLPDNEELQKI
ACA30404 Rrao         NSNIKELKEIQKQVAEIRAEVPYTFFEKLNNIWQNIKNVFVNNSEQVLAKNKESNTRTISKIDEQLYKTKHKFEELIENKERNINDIIAKLPDNEELQKI
YP_246387 Rfel        NSNIKELKEIQKQVAEIREEIPYTFFEKLNNIWQNVKNVFVNNSEQVLAKNKESNTRAIRKIDEQLYKTKHKFEELIENKERNINDIIAKLPDNEELQKI
YP_001492102 Rcan     NSNIKELKEIQKQVAEIREEIPHTFFEKLNSIWQNVKNVFINNSEQVLAKNKESNTRAIRKIEEQLYSTKHQFEKLIENQEKNINDIIGKLPDNEKLQKI
ZP_00142939 Rsib      NSNIKELKEIQKQVAEIRAEVPHTFFEKLNNIWQNVKNVFVNNSEQVLAKNKESNTRTIRKIDEQLYKTKHKFEELIENKERNIKDIIAKLPDNEKLQKI
YP_002845437 Rafr     NSNIKELKEIQKQVAEIRAEVPHTFFEKLNNIWQNVKNVFVNNSEQVLAKNKESNTRTIRKIDEQLYKTKHKFEELIENKERNIKDIIAKLPDNEKLQKI
NP_360546 Rcon        NSNIKELKEIQKQVAEIRAEVPHTFFEKLNNIWQNVKNVFVNNSEQVLAKNKESNTRTIRKIDEQLYKTKHKFEELIENKERNIKDIIAKLPDNEKLQKI
YP_001650281 Rric     NSNIKELKEIQKQVAEIRAEVPHTFFEKLNNIWQNVKNVFVNNSEQVLAKNKESNTRTIRKIDEQLYKMQHKFEELIENKERNIKDIIAKLPDNEELQKI
AEV92458 Rslo         NSNIKELKEIQKQVAEIRAEVPHTFFEKLNNIWQNVKNVFVNNSEQVLAKNKESNTRTIRKIDEQLYKTKHKFEELIENKERNIKDIIAKLPDNEKLQKI
Q9AKP3 Rmon          NSNVKELKEIQKQVAEIREEVPYTFFEKLNNIWQNVKNVFVNNSEQVLAKNKESNTRTIRKIDEQLYKTKHKFEELIENKERNINDIIAKLPDNEELQKI
                             310       320       330       340       350       360       370       380       390       400
                     ....|....|....|....|....|....|....|....|....|....|....|....|....|....|....|....|....|....|....|
Rhel AS819           VSNLTNHMTSKKEPILANSSLAKPLENNIPPPPP---LPENNIPPPP----PLPENNIPPPP----PLPENNIPPPP----PLPENNIPPPP----PLPE
WP_010421970 Rhel C9P9 VSNLTNHMTSKKEPILANSSLAKPLENNIPPPPP---LPENNIPPPP----PLPENNIPPPP----PLPENNIPPPP----PLPENNIPPPP----PLPE
YP_001493700 Rak      VSNFANHMTSKKEPILTTSSIAKPLENNITRPPL---LSQNNIPSPP----PLSKNNIPPPPP-------------------------------------
YP_001499560 Rmas     ASNLTNHMASKKEPILANSSLAKPLENNITPPPP---LSQNNIPSPPPPPPPLPENNIPP----------------------------------------
ACA30404 Rrao         VSNLTNHMALKKEPILANSSLAKPLENNITPPPP---LSQNNIPSPPPPPPPLPENNIPSPPPPPPPLPENNIPSPPPPPP---------------PLPE
YP_246387 Rfel        VSNLANHMTSKKEPILTTSSIAKPLENNVTPPPP---LTKNNIPPPPPPP-PLSKNNILPPPP-------------------------------------
YP_001492102 Rcan     VSNLADHMTAKKEPILTTSSLTKPLENNMPPPPPPPLPQNNMPPPPPPP-PLPQNNMPPP-PPPPPLPQNNMPPPPPPPPPLPQNNM------------
ZP_00142939 Rsib      VSNLTNHMASQKEPILANASLAKPLENNITPPSP---LPENNIPSPPPPPPPLPENNIPSPPPPPPPLPENNIPSPPPPPP---------------PLPE
YP_002845437 Rafr     VSNLTNHMTSQKEPILANASLAKPLENNITPPSP---LSENNIPPSPPPPPP-------PPPPPPPP------PSPP-----------------------
NP_360546 Rcon        VSNLTNHMASQKEPILANASLAKPLENNITPPSP---LPENNIPSPPPPPPPS-------------PLPENNIPSSPPPPPPP-------------PLPE
YP_001650281 Rric     VSNLTNHMASKKEPILANVSLAKPLENNIPPPPPPP--------------PLPDNNIPPPPPPPP----------------------------------
AEV92458 Rslo         VSNLTNHMASKKEPILANASLAKPLENNITPPPP---LPENNIPPPPPP--PLPENNIPPPPPP--PLPENNIPPPPPP--PLPENNIPPP---------
Q9AKP3 Rmon          VSNLTNHMASTKEPILTNSSLAKPLENNITPPPP---LPGNNIPSPPPPPPPLPGNNIPSPPPPPPPLPGNNIPSPPPPPPPLPGNNIPSPPPPPPPLSG
                             410       420       430       440       450       460       470       480       490       500
                     ....|....|....|....|....|....|....|....|....|....|....|....|....|....|....|....|....|....|....|
                                                                                                         WH2 regions
Rhel AS819           NNIPPPP----PLPENNIPPPPP--------------------------------MAP--AQTETLSKPVEATT-VKKLENQPRPSIDTSDLMKEIVGPK
WP_010421970 Rhel C9P9 NNIPPPP----PLPENNIPPPPP--------------------------------MAP--AQTETLSKPVEATT-VKKLENQPRPSIDTSDLMKEIVGPK
YP_001493700 Rak      ------------------PPPPP-----------------------------MPT-----AQTDALSKPVGVTT-VKKLENQQRPSLDTSDLMREIAGPN
YP_001499560 Rmas     ---------------------------------------------PPPPPPPMPTMAPVSAQTETLSKPVEATT-VKKPENQLRPSIDTSDLMREIVGPK
ACA30404 Rrao         NNIPSPP-------------------------------------PPPPPPPPMPTMAPASAQTETLSKPVEATT-VKKPENQPCPSIDTSDLMREIAGPK
YP_246387 Rfel        --------------------------------------------------PPMPTMAP--AQTETLSKPVGVTTTVKKLENQPRPSIDTSDLMREIAGPK
YP_001492102 Rcan     ----------------------------------------------PPPPPPMPTMAP--AQTETLLKSIEATT-VKNPENQSHPSIDTSDLMKEIVGPR
ZP_00142939 Rsib      NNIPSPPP----------------------------------------PPPPPPPMAP----AETLSKPVESTT-VKKLENQPRPSIDTSDLMREIAGPK
YP_002845437 Rafr     ------------------------------------------------PPPPPPPMAP--AQAETLSKPVESTT-VKKLENQPRPSIDTSALMREIAGPK
NP_360546 Rcon        NNIPSPPPPP--------------------------------------PPPPPPPMAP--AQAETLSKPIESTT-VKKLANQPRPSIDTSDLMREIAGPK
YP_001650281 Rric     -----------------------------------------------------PPMAPV----KTLSKAVEATT-VKKLENQPRPSIDTSDLMREIAGPK
AEV92458 Rslo         ------------------------------------------------PPPPPPPM----AQAETLSKPVESTT-VKKLENQPRPSIDTSDLMREIVGPK
Q9AKP3 Rmon          NNIPSPPPPPPPLSGNNIPSPPPPPPPLPGNNIPSPPPPPPPLSQNNIPPPPPPPMAPVSAQTEKLSKPVEATT-VKKPENQPRPSIDTSDLMREIAGPK
                             510       520       530       540       550       560       570       580       590       600
                     ....|....|....|....|....|....|....|....|....|....|....|....|....|....|....|....|....|....|....|
                                                                                 central region       acidic domain
Rhel AS819           NLRKVEKTDVKAQDSRDLLLQSIRGEHKLKKVEFDPNTGKPVAYSHSKPAQNVSKPNEIASILARRVAMEMSDSSSSS-SGSESDSGNWSDVST---KPK
WP_010421970 Rhel C9P9 NLRKVEKTDVKAQDSRDLLLQSIRGEHKLKKVEFDPNTGKPVAYSHSKPAQNVSKPNEIASILARRVAMEMSDSSSSS-SGSESDSGNWSDVST---KPK
YP_001493700 Rak      NLRKVEKTDVKIQDSRDLLLQSIRGEHKLRKVAFDPNTGKPVAHSHSKPAKNVNQPNGVASILARRVAMEMSDSSSS-GSESDSDSGNWSDISVNSDKPK
YP_001499560 Rmas     KLRKVEKTDVKAQDSRDLLLQSIRGEHKLKKVEFDPNTGKPVAHSHSKPAQNINKPNGVASILARRVAIEMSDSSSSSGSES--DSGNWSDVSVNRNKSK
ACA30404 Rrao         KLRKVEKTDVKAQDSRDLLLQSIRGEHKLKKVEFDPNTGKPVAHSHSKPAQNVNKPNGIASILARRVAMEMSDSSSSSGSES--DSGNWSDVSVNRNKSK
YP_246387 Rfel        NLRKVEKTDVKTQDSRDLLLQSIRGEHKLRKVEFDPNTGKPVAHSHSKPAQNVSKPNGVASILARRVAMEMSDSSSSSGSES--DSGNWSDASVNSNKPK
YP_001492102 Rcan     NLKEVKKIDAKAQDPRDLLLQSIRGEHKLKKVEFDPN-GRPVAHSRSKPAQNVNKSNEIVEILARRVAMEMSSSGS----ESDSDSGNWSDVST---KSK
ZP_00142939 Rsib      ---------------------------KLKKVEFDPNTGKPVAHSHSKPAQNVNKPSGLESIFARRAAIEVSDSSS---SESESDSGNWSDVSVNRNKSK
YP_002845437 Rafr     ---------------------------KLKKVEFDPNTGKPVVHSHSQPAQDVNKPSGLESILARRIAIEVSDSSS---SES--DSGNWSDVSVNRNKSK
NP_360546 Rcon        ---------------------------KLKKVEFDPNTGKPVAHSHSKPAQNVNALSGLESIFARRAVIKVSDSSS---SES--DSGNWSDVSVNRNKSK
YP_001650281 Rric     ---------------------------KLKKVEFDPNTGKPVAHSHSKPAQNVNKPSGLESIFARRVAIEMSDSSS---SES--DSGNWSDVSVNRNKSK
AEV92458 Rslo         ---------------------------KLKKVEFDPNTGKPVTHSHSKPAQNVNKPSGLESIFARRFAIEVSDSSS---SES--DSGNWSDASVNRNKSK
Q9AKP3 Rmon          KLRKVEETDVKVQDSRDLLLQSIRGEHKLKKVEFDPNTGKPVAHSHSKPVQNVNKLSGVASILARRVVMEMSDSSG---SES--DSGNWSDVGVNRN-TK
                                                                       Conserved sequence ΦXXΦXXΦXXXRXXΦ
```
