# Supplementary material for: In vitro studies of Rickettsia-host cell interactions: Confocal laser scanning microscopy of Rickettsia helvetica-infected eukaryotic cell lines
Source: PLoS Negl Trop Dis. 2018 Feb 12;12(2):e0006151. doi: 10.1371/journal.pntd.0006151 (PMC5825168; doi:10.1371/journal.pntd.0006151)
Supplement: S2 Fig — Numbering above the alignment indicates amino acid numbering according to R. conorii Sca2 (accession number NP 359747). Dashes within the alignment indicate deletions. * indicate stop-codons. Amino acids 309 to 347, which have been described necessary for the stimulation of actin assembly by Sca2, are shaded in grey; WH2 domains are shaded in blue, and proline-rich domains are boxed in black. Abbreviations: Rcon—R. conorii, Rric—R. rickettsii, Rpar—R. parkeri, Rpea–R. peacockii, Rhel—R. helvetica. (PDF) [file pntd.0006151.s002.pdf]

```
                              ....|....| ....|....| ....|....| ....|....| ....|....|
                                     10         20         30         40         50
NP 359747 Rcon                MNLQNSHSKK YVLTFFMSTC LLTSSFLSTS ARAASFKDLV SKTPAWEKHN
AAT79547 Rric                 ---------- ---------- ---------- ---ASFKDLV SKTPAWAKHN
ADH59734 Rpar                 ---------- ---------- ---------- ---ASFKDLV SKTPAWEKHN
YP_002916397 Rpea             ---------- ---------- ---------- ---ASFKDLV SKTPAWEKHN
Rhel AS819                    MNLQNSHSKK YVLTFFMSTY LLTSSFLSTS ARAASFKDLV SKTPAWEKHN

                              ....|....| ....|....| ....|....| ....|....| ....|....|
                                     60         70         80         90        100
NP 359747 Rcon                STQQQNIWKD LTPNEKIKKW QEAALVPSFT QAQNDLGIKY KETDLSSFLD
AAT79547 Rric                 STQQQNIWKD LTPTEKIKKW QEAALVPSFT QAQNDLGIKY KETDLSSFLD
ADH59734 Rpar                 STQQQNIWKD LTPNEKIKKW QEAALVPSFT QAQNDLGIKY KETDLSSFLD
YP_002916397 Rpea             ATQQQNIWND LTQNEKIKKW QEAALVPSFT QAHNDLGIKY KETDLSSFLD
Rhel AS819                    SKQQ*NIWKD FTPNEKIKKW QEADLIPSFS QAQDDLGIKY KETDLSSFLD

                              ....|....| ....|....| ....|....| ....|....| ....|....|
                                    110        120        130        140        150
NP 359747 Rcon                NTRHKARQAR AEILLYIERV KQQDFDTKKQ AYINQGVVPT DIEAATNLGI
AAT79547 Rric                 NTRHKARQAR AEILLYIERI KQQDFDTKKQ AYINQGVVPT DIEAATNLGI
ADH59734 Rpar                 NTRHKARQAR AEILLYIERV KQQDFDTKKQ EYINQGVVPT DIEAATNLGI
YP_002916397 Rpea             NTRHKARQAR AKILLYIERV KQQDFDTKKQ EYINQGVVPT DIEAATNLGI
Rhel AS819                    NTRHKARQAR AKILLYIERV KQQDFDTKKQ EYIKQGVVPT DMEAATNLGI

                              ....|....| ....|....| ....|....| ....|....| ....|....|
                                    160        170        180        190        200
NP 359747 Rcon                SYDPSKIDNN VEHDQKVRRA EKDKKAVIEL YVSSINRGIK YKHYVDNDII
AAT79547 Rric                 SYDPSKIDHN VEHDQKVRRA EKDKKAVIEL YISSINRDIK YKHYVDNDII
ADH59734 Rpar                 SYDPSKIDNK VEHDQKVRRA EKDKKAVIEL YISSINRGIK YKHYVDNDII
YP_002916397 Rpea             SYDPSKIDNN VEHDQKVRRA EKDKKAVIEL YISSINRDIK YKHYVDNDII
Rhel AS819                    SYDPSKIDNN IEKDQKVRRA EKDKKAVIDL YISSINRDIK YKHYVDNDII

                              ....|....| ....|....| ....|....| ....|....| ....|....|
                                    210        220        230        240        250
NP 359747 Rcon                PEIQEVRTAL NMNKDDAQSF VASIRTEIME NAKGQYIADS HIPTEKELKK
AAT79547 Rric                 PEMQEVRTAL NMNKDDAQSF VASIRTEIME NAKGQYIADS HIPTEKELKK
ADH59734 Rpar                 PEMQEVRTAL NMNKDDAQSF VASIRTEIME NAKGQYIADS HIPTEKELKK
YP_002916397 Rpea             PELQEVRTAL NMNKDDAQSF VASIRTEIME NAKGQYIADS HIPTEKELKK
Rhel AS819                    PKMKEVRTAL NINKDDAESF IGSIRTEIME NAKG*YIADS LFLQKKS*SR

                              ....|....| ....|....| ....|....| ....|....| ....|....|
                                    260        270        280        290        300
NP 359747 Rcon                KFGISRDDNR DGYIKSIRLK VMDKEKPQYI ADSHIPTEKE LEQKFGADKG
AAT79547 Rric                 KFGISRDDNR DGYIKSIRLK VMENAKGQYI ADSHIPTEKE LEQKFGADKG
ADH59734 Rpar                 KFGISRDDNR DGYIKSIRLK VMDKEKPQYI ADSHIPTEKE LEQKFGVDKG
YP_002916397 Rpea             KFGISRDDNR DGYIKSIRLK VMDKEKPQYI ADNHIPTEKE LEQKFGADKG
Rhel AS819                    NLVLIKVRQQ TISHQ*KLR* CLVKNRTI*I IILSLTLTN* *MNLR*GK*K

                              ....|....| ....|....| ....|....| ....|....| ....|....|
                                    310        320        330        340        350
NP 359747 Rcon                EATNYIASIA TQMMLDKKSY YIDNNIIPNA DELMNEFKIG PVKATSYINQ
AAT79547 Rric                 EATNYIASIA TQMMLGKKSY YIDNNIIPNT DELMNEFNIG PVKATSYINQ
ADH59734 Rpar                 EATNYIASIA TQMMLGKKSY YIDNNIIPNA DELMNEFKIG PVKATSYINQ
YP_002916397 Rpea             EATNYIAS-- ---------- ---------- ---------- -------INQ
Rhel AS819                    QILI*NK*EP E*NQFLNNND TTKPSALDAL KKRAGPKVII GICQIKVQII

                              ....|....| ....|....| ....|....| ....|....| ....|....|
                                    360        370        380        390        400
NP 359747 Rcon                IRAGIEANQF LNNNDTTKPS TGRSQKKSGS KNDHWYMSNQ SINNTGTSAR
AAT79547 Rric                 IRAGIEAKQF LNNNDTTKPS TGHSQKKSGS KNDHWYMSNQ SINDTGTSSR
ADH59734 Rpar                 IKAGIEANQF LNNNDTTKPS TGRSQQKSGS KNNPWYMSNQ SIHNTETSSQ
YP_002916397 Rpea             IRAGIEANQF LNNNDTTKPS TGRSQKKSGS KNDHWYMSNQ SINDTRTSSR
Rhel AS819                    QEHLRGCPEK RNNHILLIL* VLLKLTLIIK KVRVI*LKLN TI*IE*YNKK

                              ....|....| ....|....| ....|....| ....|....| ....|....|
                                    410        420        430        440        450
NP 359747 Rcon                IVTGREKKQR YFFDPISTFK TYFNTKASKG NLTQSQHNIN RIIQQEENIE
AAT79547 Rric                 IFTGREKKQR YFFDPISTFK THFNTKASKG NLTQSQHTIK RIIQQEENIA
ADH59734 Rpar                 ISTGRDKKQR YFFDSISTFK TQFNAKANKG NLTLSQQNIN KLIEQEENIE
YP_002916397 Rpea             IFTGRAKKQR YFFDPISTFK THFNTKANKG NLTQSQHNIN RIIQQEENIE
Rhel AS819                    KNIEEFKNLI KTDPVAALNL KVDSSYKKEA VNTIFSDFND DTIQKVLFSN
```

```
                        ....|....| ....|....| ....|....| ....|....| ....|....|
                                460        470        480        490        500
NP 359747 Rcon          EFKNLIKTDP IAALTLQVDS SYKQEAVTTI LSDFNDDTIQ RVLFSNDKGK
AAT79547 Rric           EFKNLIKTDP IAALTLQVGS SYKQEAVTTI LSDFNDNTIQ RVLFSNDKGQ
ADH59734 Rpar           QFKDLIKTNP IAALLLQVDS SYKNQAVKII LKDRNDNTIQ RLLFTNDTGQ
YP_002916397 Rpea      EFKNLIKTDP IAALTLQVDS SYKQEAVTTI LSDFNDDTIQ RVLFSNDKGQ
Rhel AS819              DKE*LDFNTN IDVKNRPILK ELLENSSSEE KTKFAERIKD YATRNISNSQ

                        ....|....| ....|....| ....|....| ....|....| ....|....|
                                510        520        530        540        550
NP 359747 Rcon          LDFNTNIDVK NRPILQELLE NSSSEEKTKF AERIQDYATR NISNSQFEEK
AAT79547 Rric           LDFKTNIDVK NRPILQELLE NSSSEEKTKF AERIQDYATR NISHSQFEEK
ADH59734 Rpar           LDFNTNIKVK NRPILQTFLN NSTSKDKTKF AEIIQDYATR NISNSQFEEK
YP_002916397 Rpea      LDFKTNIDVK NRPILQELLE NSSSEEKTKF VERIQDYATR NISNSQFEEK
Rhel AS819              FEEKARLDFI KLAASKDKSS VEKFLALQLE L*NKMQPHII KSVYILTSEI

                        ....|....| ....|....| ....|....| ....|....| ....|....|
                                560        570        580        590        600
NP 359747 Rcon          ARLDLIKLAA SKDKSSVENF LTLQLELKNR MQPYVVNSVY ILTPEIVKEI
AAT79547 Rric           ARLDLIKLAA SKDKRSVENF LTLQLELKNR MQPYIVNSAY ILTPEIVKEI
ADH59734 Rpar           ARLDLIKLAA SKDKSSVENF LTLQLELKNR MQPYIVNSVY ILTPEIVKEI
YP_002916397 Rpea      ARLDLIKLAA SKDKSSVENF LTLQLELKNR IQPHIANSVY VLTLEMVKEI
Rhel AS819              VEEINIELKN KGLIIDSLTK DDMIKLAKEV NKQTLNSVIK VILSDSNVLS

                        ....|....| ....|....| ....|....| ....|....| ....|....|
                                610        620        630        640        650
NP 359747 Rcon          NIELKNKGLI RDSLTKDYMI KLAKEVNNHT LNSVIKVILS DSKILSNETN
AAT79547 Rric           NIELKNKGLI RDSLTKDYMI KLAKEVNNHT LNSVIKVILS DSNILSNETN
ADH59734 Rpar           NIELKNKGLI RDSLTKDYMI KLAKEVNNHT LNSVIKVILS DSNILSNEAN
YP_002916397 Rpea      NIELKNKGLI KDSLTKDYMI KLAKEINNHT LNSVIKVILS DSNILSNETN
Rhel AS819              NETNKILGLA VGNNTNNLEQ TQSGIPNPPP LPLNGSMPPP PPPLNSQGFS

                        ....|....| ....|....| ....|....| ....|....| ....|....|
                                660        670        680        690        700
NP 359747 Rcon          KILGLAVSNN ANNLEQTQSG IPNPPPLPLN GGIPNPPPLP LNGSMPPPPL
AAT79547 Rric           KILGLAVGNN ANNLEQTQSG IPNPPPLPLN G......... ..........
ADH59734 Rpar           KILGLAVGNN ANNLEQTQSD IPNPPPLPLN G......... ..........
YP_002916397 Rpea      KILGLAVGNN ANNLEQTQSG IPNPPPLPLN G......... ..........
Rhel AS819              SNSNNFDLNK LQAEYTQFTR NTTVQLKAPL QPLVQPAQKE ASQRQLMPNY

                        ....|....| ....|....| ....|....| ....|....| ....|....|
                                710        720        730        740        750
NP 359747 Rcon          HSQGFSSNSK HFDLNQLQTE YPHIHSLYVQ FTHNTTVQSK APLQPTASSA
AAT79547 Rric           .......... .......... .......... .......... ..........
ADH59734 Rpar           .......... .......... .......... .......... ..........
YP_002916397 Rpea      .......... .......... .......... .......... ..........
Rhel AS819              I*NIELRQEV KKLMIYKISL *KENMTLQML YVKY*TELYA NQGADEKTLV

                        ....|....| ....|....| ....|....| ....|....| ....|....|
                                760        770        780        790        800
NP 359747 Rcon          TSTGRSTPET AYAKLYAEYR TETGGTKAND LQDQLIKRQA DLTNVIRQIL
AAT79547 Rric           .......... .......... .......... .......... ..........
ADH59734 Rpar           .......... .......... .......... .......... ..........
YP_002916397 Rpea      .......... .......... .......... .......... ..........
Rhel AS819              NLFSISTLEI EEKAKEAFNK LVQDPYIQDI TVNGKKATTS EEILKNLFNE

                        ....|....| ....|....| ....|....| ....|....| ....|....|
                                810        820        830        840        850
NP 359747 Rcon          TESYANQGAD EKTLLNLFSI STPEIAEKAK EAFNTLAQDQ YIKDITVNGK
AAT79547 Rric           .......... .......... .......... .......... ..........
ADH59734 Rpar           .......... .......... .......... .......... ..........
YP_002916397 Rpea      .......... .......... .......... .......... ..........
Rhel AS819              DTDDAVKRIL LSSCKNI*RI KETY*T*IKY *KLLKSTRKK KLKNTSIPET

                        ....|....| ....|....| ....|....| ....|....| ....|....|
                                860        870        880        890        900
NP 359747 Rcon          KTITSEEIIK NLFNEDTDDA IKRILLSSCK ISEELKRPIK LEFNKSELIR
AAT79547 Rric           .......... .......... .......... .......... ..........
ADH59734 Rpar           .......... .......... .......... .......... ..........
YP_002916397 Rpea      .......... .......... .......... .......... ..........
Rhel AS819              CHHCHRLVQK IWN*LI**V* GLLKIG*VEL LN*TQIHLLL K*LQKFITLA
```

```
                          ....|....| ....|....| ....|....| ....|....| ....|....|
                                 910        920        930        940        950
NP 359747 Rcon            ELQGKQNPFK QLEFAYINTK NFDQDIFGNR IDELINNPNI LTIVQQATFL
AAT79547 Rric             .......... .......... .......... .......... ..........
ADH59734 Rpar             .......... .......... .......... .......... ..........
YP_002916397 Rpea         .......... .......... .......... .......... ..........
Rhel AS819                PI*L*DIKNL LYQDKNQQDI KQNTLMPIYL KSYNGISSI* AFKKFI*SSS

                          ....|....| ....|....| ....|....| ....|....| ....|....|
                                 960        970        980        990       1000
NP 359747 Rcon            TKEDTNLRKT INSDQAQAKL DDLRTAILST IKIEELITAN LPQHDFIAIV
AAT79547 Rric             .......... .......... .......... .......... ..........
ADH59734 Rpar             .......... .......... .......... .......... ..........
YP_002916397 Rpea         .......... .......... .......... .......... ..........
Rhel AS819                K**VFCKNPR SSQHYAL*LY RS*N*NRARS T*YL*IQTFR VSKR*RIVFI

                          ....|....| ....|....| ....|....| ....|....| ....|....|
                                1010       1020       1030       1040       1050
NP 359747 Rcon            KEKDPELLKE FLKATTLTVT GNNNLDQLRL ALPSFTGMSN EQIRILSNKL
AAT79547 Rric             .......... .......... .......... .......... ..........
ADH59734 Rpar             .......... .......... .......... .......... ..........
YP_002916397 Rpea         .......... .......... .......... .......... ..........
Rhel AS819                C*TITSRVY* TRTKIR**CS IRK**QRKAS RTFRSETITI IPTRKRSYK*

                          ....|....| ....|....| ....|....| ....|....| ....|....|
                                1060       1070       1080       1090       1100
NP 359747 Rcon            KMSIILKALK ECSQEKATQY IHTGNMPPPP PPPPPLPDSQ DLELAYLKSL
AAT79547 Rric             .......... .......... .......... .......... ..........
ADH59734 Rpar             .......... .......... .......... .......... ..........
YP_002916397 Rpea         .......... .......... .......... .......... ..........
Rhel AS819                RILNKGLYTT *R*Q*KVRNI GFKNHFINDV IKF*R*RKL* IIKMTKDHFW

                          ....|....| ....|....| ....|....| ....|....| ....|....|
                                1110       1120       1130       1140       1150
NP 359747 Rcon            GITKANTSTF KTTPKTYHFS SDIALRYKEF TLSGQKSAGY KAKYSDADLL
AAT79547 Rric             .......... .......... .......... .......... ..........
ADH59734 Rpar             .......... .......... .......... .......... ..........
YP_002916397 Rpea         .......... .......... .......... .......... ..........
Rhel AS819                H*DLVMKKLV *TEGYGLAAY TVSTNKELGK IFQNIKVVPP A*LSGLMLNL

                          ....|....| ....|....| ....|....| ....|....| ....|....|
                                1160       1170       1180       1190       1200
NP 359747 Rcon            KKAIVESVAF EHSKNLSKAH QNNKYFEQIQ KAVNTMYSSF IGHRTELEQK
AAT79547 Rric             .......... .......... .......... .......... ..........
ADH59734 Rpar             .......... .......... .......... .......... ..........
YP_002916397 Rpea         .......... .......... .......... .......... ..........
Rhel AS819                SIVMML*E*L IAVWNPKLNI IKN*EKQQLT VIF*AFTV*K H*LKVSRCRL

                          ....|....| ....|....| ....|....| ....|....| ....|....|
                                1210       1220       1230       1240       1250
NP 359747 Rcon            IHNIYTSKLL ELTKDKEFIK YVEDNIILNK KLTKAFTSAD SDFIDSRTEL
AAT79547 Rric             .......... .......... .......... .......... ..........
ADH59734 Rpar             .......... .......... .......... .......... ..........
YP_002916397 Rpea         .......... .......... .......... .......... ..........
Rhel AS819                *HLTVIIILR INLKVLII*L ENIKTII*VL KAR*IINIV* NMIYI*SLIS

                          ....|....| ....|....| ....|....| ....|....| ....|....|
                                1260       1270       1280       1290       1300
NP 359747 Rcon            EQKIHNIYIQ QLTKYPEEEV KEAFNTASLD FIGPRTEIGQ EVHNIYKSQL
AAT79547 Rric             .......... .......... .......... .......... ..........
ADH59734 Rpar             .......... .......... .......... .......... ..........
YP_002916397 Rpea         .......... .......... .......... .......... ..........
Rhel AS819                VLNMIIQELA TIKSII*I*K I**FKRNQIN HLKVVLAVK* CLNL*RLQVI

                          ....|....| ....|....| ....|....| ....|....| ....|....|
                                1310       1320       1330       1340       1350
NP 359747 Rcon            LELTKDTELC LFTQQVLAEA TELEQKYGSD IQSENSNNEK KVERLDQEKL
AAT79547 Rric             .......... .......... .......... .......... ..........
ADH59734 Rpar             .......... .......... .......... .......... ..........
YP_002916397 Rpea         .......... .......... .......... .......... ..........
Rhel AS819                *Y*HQVCTVI LSITSIIKIQ KLMQKRLLKD KHYKKQLLLY QNNLSLDIIS
```

```
                       ....|....| ....|....| ....|....| ....|....| ....|....|
                               1360       1370       1380       1390       1400
NP 359747 Rcon         QLFKQENEAT NDESSTKDDT QPEDSNKKSE QSDSKTALSP RLLSSNDSKN
AAT79547 Rric          .......... .......... .......... .......... ..........
ADH59734 Rpar          .......... .......... .......... .......... ..........
YP_002916397 Rpea      .......... .......... .......... .......... ..........
Rhel AS819             VVIYL*VEKI *MFYLSIIII HIENIKAIRD LLS*KLICKA ITQPL*DFSL

                       ....|....| ....|....| ....|....| ....|....| ....|....|
                               1410       1420       1430       1440       1450
NP 359747 Rcon         DKSSDDKKSL LALRSSDEDD TGYATDEEEL EESNSTTDEE LKKDVVLESE
AAT79547 Rric          .......... .......... .......... .......... ..........
ADH59734 Rpar          .......... .......... .......... .......... ..........
YP_002916397 Rpea      .......... .......... .......... .......... ..........
Rhel AS819             LVVEYTSIQL GK*QGVCKPR SGAYIKRSAS EDLQRHCNQF FKLY*LGVDL

                       ....|....| ....|....| ....|....| ....|....| ....|....|
                               1460       1470       1480       1490       1500
NP 359747 Rcon         DEAIDVSFKT EAITEQDEVT QRQQVSDDTS GKVAILVQAT STLHKPVHYN
AAT79547 Rric          .......... .......... .......... .......... ..........
ADH59734 Rpar          .......... .......... .......... .......... ..........
YP_002916397 Rpea      .......... .......... .......... .......... ..........
Rhel AS819             TVRSCWVQKK RYTRTRQKFN NL*KKETVR* QYTNKK**SQ ELVYYN*QNN

                       ....|....| ....|....| ....|....| ....|....| ....|....|
                               1510       1520       1530       1540       1550
NP 359747 Rcon         INDRLTVAAI GAGDEETSIN RGVWISGLYG INKQRIWKNI PKYQNRTTGI
AAT79547 Rric          .......... .......... .......... .......... ..........
ADH59734 Rpar          .......... .......... .......... .......... ..........
YP_002916397 Rpea      .......... .......... .......... .......... ..........
Rhel AS819             *VTYRKHVK* WVIAEIHFTA IKSFMNKVEK KRYMR*AEES LV*RTEYQQV

                       ....|....| ....|....| ....|....| ....|....| ....|....|
                               1560       1570       1580       1590       1600
NP 359747 Rcon         TIGTDAEFIN SHDVIGIAYS RLESQIKYNK KLGKTTVNGH LLSIYSLKEL
AAT79547 Rric          .......... .......... .......... .......... ..........
ADH59734 Rpar          .......... .......... .......... .......... ..........
YP_002916397 Rpea      .......... .......... .......... .......... ..........
Rhel AS819             LKML*LILQL NIPLMVKNVQ LMSYARKE*L FQEEA*DQFG FVMI*KILRN

                       ....|....| ....|....| ....|....| ....|....| ....|....|
                               1610       1620       1630       1640       1650
NP 359747 Rcon         IKGFSLQTIT SYGHNYIKNR SKNINNIIGK YQNNSLSFQT LLNYKYRTKY
AAT79547 Rric          .......... .......... .......... .......... ..........
ADH59734 Rpar          .......... .......... .......... .......... ..........
YP_002916397 Rpea      .......... .......... .......... .......... ..........
Rhel AS819             A*KL*KIR*Q ATVLF*MIYS *QLLKSKN.. .......... ..........

                       ....|....| ....|....| ....|....| ....|....| ....|....|
                               1660       1670       1680       1690       1700
NP 359747 Rcon         DLHFIPNIGF QYDYSRASNY KEYNVDIENL MIQKKSNQLF ESSLGGKIVF
AAT79547 Rric          .......... .......... .......... .......... ..........
ADH59734 Rpar          .......... .......... .......... .......... ..........
YP_002916397 Rpea      .......... .......... .......... .......... ..........
Rhel AS819             .......... .......... .......... .......... ..........

                       ....|....| ....|....| ....|....| ....|....| ....|....|
                               1710       1720       1730       1740       1750
NP 359747 Rcon         KPIVTTNNIV LTPSLYGNIE HHFNNKNTKV NAKATFKGQT LQETIITLKQ
AAT79547 Rric          .......... .......... .......... .......... ..........
ADH59734 Rpar          .......... .......... .......... .......... ..........
YP_002916397 Rpea      .......... .......... .......... .......... ..........
Rhel AS819             .......... .......... .......... .......... ..........

                       ....|....| ....|....| ....|....| ....|....| ....|
                               1760       1770       1780       1790
NP 359747 Rcon         PKLGYNIGSN ILMSRKNINV LLEYNYYTHR KYQSHQGLIK LKVNL
AAT79547 Rric          .......... .......... .......... .......... .....
ADH59734 Rpar          .......... .......... .......... .......... .....
YP_002916397 Rpea      .......... .......... .......... .......... .....
Rhel AS819             .......... .......... .......... .......... .....
```
